# Vitamin D Supplementation during Intensive Care Unit Stay Is Associated with Improved Outcomes in Critically Ill Patients with Sepsis: A Cohort Study

**DOI:** 10.3390/nu15132924

**Published:** 2023-06-28

**Authors:** Boshen Yang, Yuankang Zhu, Xinjie Zheng, Taixi Li, Kaifan Niu, Zhixiang Wang, Xia Lu, Yan Zhang, Chengxing Shen

**Affiliations:** 1 Department of Cardiology, Shanghai Sixth People’s Hospital Affiliated to Shanghai Jiao Tong University School of Medicine, Shanghai 200235, China; yangboshen323@sjtu.edu.cn (B.Y.); litaixi01@163.com (T.L.); nkf1997@163.com (K.N.); wang_zhixiang2022@126.com (Z.W.); xialu0292@163.com (X.L.); 2 School of Medicine, Shanghai Jiaotong University, Shanghai 200030, China; zyk3336@163.com; 3 International Institutes of Medicine, Zhejiang University School of Medicine, Yiwu 310030, China; zhengxinjie@zju.edu.cn; 4 Shanghai Institute for Biomedical and Pharmaceutical Technologies, Shanghai 201203, China

**Keywords:** vitamin D, sepsis, intensive care units, mortality risk, prognosis

## Abstract

Background: Vitamin D, as a common micronutrient, has been widely used in critically ill patients. However, whether supplementation of vitamin D in adult patients with sepsis can improve their prognosis remains controversial. Methods: Data from the Mart for Intensive Care IV database was used in this retrospective cohort study, and adult patients with sepsis were enrolled. Critically ill patients, admitted to intensive care units (ICUs) between 2008 and 2019 at the Beth Israel Deaconess Medical Center (BIDMC), were divided into the vitamin D supplementation group and non-vitamin D supplementation group. The primary outcomes were defined as all-cause in-hospital, 28-day, and 90-day mortality rates after admission to the ICU. A 1:1 propensity score matching (PSM), inverse probability of treatment weighting (IPTW), and overlap weighting (OW) analyses were used to minimize selection bias and balance the baseline demographic characteristics. Regression and survival analyses were performed to assess the association between vitamin D supplementation and clinical outcomes in patients with sepsis. Results: In total, 3539 patients with sepsis were enrolled as study participants; of these, 315 were supplemented with vitamin D during their ICU stay. In-hospital, 28-day, and 90-day mortality rates were significantly lower in patients with sepsis supplemented with vitamin D. Multivariate regression analysis showed vitamin D supplementation as a potential protective factor for in-hospital mortality with an odds ratio (OR) = 0.70 (0.51–0.96) after adjusting for all confounders. The hazard ratios (HRs) for 28-day and 90-day mortality were 0.65 (0.50–0.85) and 0.70 (0.55–0.90), respectively. The survival analysis showed that the vitamin D supplementation group had a higher survival probability within 28 and 90 days (*p*-value < 0.05). These results remained relatively stable post PSM, IPTW, and OW. However, we found no evidence that vitamin D supplementation could shorten the length of stay in the ICU or hospital. Conclusions: Vitamin D supplementation during an ICU stay was associated with improved prognosis in patients with sepsis, as evidenced by lower in-hospital, 28-day, and 90-day mortality rates and lower disease severity-related scores, but showed no influence on the length of stay in the hospital or ICU.

## 1. Introduction

Vitamin D deficiency, arising from insufficient sunlight exposure or low dietary intake, has been a non-negligible risk factor for autoimmune and infectious diseases [1]. Sepsis is characterized by dysfunctional infection responses and excessive systemic inflammation [2], and is a leading contributor to mortality in intensive care units (ICUs) [3]. The association between vitamin D deficiency and sepsis has been recognized for a long time. Vitamin D deficiency, prevalent in critically ill populations diagnosed with sepsis [4], is related to an increased risk of sepsis and worse prognosis in patients with sepsis [5,6,7,8]. Moreover, previous studies have demonstrated that patients with lower serum vitamin D levels had a longer length of hospital stay [9] and might have an independent relationship with the duration of mechanical ventilation (MV) in critically ill children [10]. 

To date, there have been no clinical practice guidelines for vitamin D supplementation in critically ill patients [11]. In principle, adequate vitamin D is an essential factor in the maintenance of musculoskeletal health, and is considered as the first step in the treatment of patients with osteoporosis [12], especially for people aged 50 years or older [13]. Although randomized controlled trials (RCTs) have shown that postmenopausal women and patients with acute respiratory tract infections can benefit from vitamin D supplementation [14,15], no beneficial effects were observed in patients with cardiovascular and metabolic diseases, even those with low baseline vitamin D levels [16]. Thus, it is necessary to identify an appropriate population who is more likely to benefit from vitamin D supplementation. 

Vitamin D is involved in the process of immunomodulation, especially in the context of autoimmunity [17]. Vitamin D has multiple pharmacological effects, such as alleviating the inflammatory response, promoting phagocytosis, and inducing lymphocyte proliferation [18]. Animal experiments have shown that vitamin D alleviated acute lung injury induced by sepsis via downregulation of ER stress [19] and the strong anti-sepsis effect was related to the upregulation of vitamin D receptor expression [20]. Therefore, vitamin D supplementation for critically ill patients with sepsis is an attractive strategy in clinical practice. 

Previous RCTs and meta-analyses have reported that supplementation of vitamin D reduced the incidence of septic shock in children with sepsis [21], decreased the duration of MV and ICU stay [22], and reduced the mortality rate among critically ill patients [23]. However, some researchers have reported disappointing results as higher mortality rates were observed in vitamin D administration groups among critically ill patients [24,25]. Up to now, there is no consensus on whether critically ill patients can benefit from vitamin D supplementation. Moreover, most studies did not focus on specific patients with sepsis, and little is known about whether adult patients with sepsis can benefit from vitamin D supplementation to improve their prognosis. This is the first study to explore the association between vitamin D supplementation and clinical outcomes in patients with sepsis admitted to ICUs among the American population.

The variability in existing results may be due to the heterogeneity of the critically ill population. Hence, in this study, we aimed to explore whether patients with sepsis admitted to ICUs between 2008 and 2019 at the Beth Israel Deaconess Medical Center (BIDMC), can benefit from the supplementation of vitamin D to improve their prognosis. 

## 2. Materials and Methods

### 2.1. Data Source

The Mart for Intensive Care IV (MIMIC-IV) database is a high-quality and large-scale database that includes data of critically ill patients admitted to ICUs between 2008 and 2019 at the BIDMC [26]. Different types of clinical data, including demographics, vital signs, laboratory data, hospital and ICU admission and discharge times, medications, and nursing records of each patient have been documented in this database. One of our team members passed the Examination of Protection of Human Research Participants and was able to access the database. PgAdmin4 and structured query language were used to mine data from the database.

### 2.2. Study Design and Participants

This is a retrospective observational study based on a publicly available database. For patients with more than one hospital admission and ICU admission records, only the first ICU stay in the first hospital admission was included for analysis. The inclusion criteria were as follows: (1) diagnosed with sepsis at hospital admission and (2) age ≥ 18 years old. The exclusion criterion was patients with no ICU stay record. Patients were divided into a vitamin D supplementation group and non-vitamin D supplementation group based on whether they were supplemented with vitamin D during their stay in the ICU. Vitamin D tablets were used for supplementation in patients with sepsis in fractions. The route for vitamin supplementation was PO or PO/NG. PO represents an oral administration, while NG represents nasogastric feeding. Based on the Endocrine Society Clinical Practice Guideline [27] and the vitamin D intake of each patient during the ICU stay, we further divided the vitamin D supplementation group’s patients into three sub-groups: (1) low-dose group: vitamin D intake < 800 IU; (2) moderate-dose group: 800 IU ≤ vitamin D intake < 2000 IU; and (3) high-dose group: vitamin D intake ≥ 2000 IU. Patients who were treated with vitamin D before admission to ICU, but the administration was stopped within 24 h after admission to the ICU, were included in the non-vitamin D supplementation group.

### 2.3. Variable Extraction

The patients’ baseline characteristics were extracted and analyzed to avoid potential confounders. The baseline characteristics were as follows: demographic data, including age, sex, and weight; vital signs, such as respiratory rate (RR), temperature, blood pressure including systolic blood pressure (SBP) and diastolic blood pressure (DBP); comorbidities, including myocardial infarction (MI), atrial fibrillation (AF), chronic heart failure (CHF), acute kidney injury (AKI), chronic kidney disease (CKD), diabetes, osteoporosis, septic shock, and cerebral diseases; clinical indices, including red blood cell (RBC), white blood cell (WBC), hemoglobin, platelet, creatinine, glucose, lactate, potassium, and chloride; and clinical measures, including vasopressin medication, antibiotic medication, and mechanical ventilation (MV). Comorbidities were diagnosed at hospital admission. The first measurements of vital signs and clinical indices within 24 h after entering the ICU were used as baseline characteristics. Indices with missing values (>30%) were deleted, and the rest was supplemented with multiple imputations.

### 2.4. Clinical Outcomes

The primary outcomes were defined as all-cause in-hospital mortality, 28-day mortality, and 90-day mortality rates after admission to ICU. The secondary outcomes were mean values of the Acute Physiology Score (APS) III, Sequential Organ Failure Assessment (SOFA), and Simplified Acute Physiology Score (SAPS) II scores during the ICU stay. The mean values of SAPS II, SOFA, or APS III were calculated based on the maximum and minimum values of SAPS II, SOFA, or APS III for each patient during the ICU stay. Additionally, the length of stay (LOS) in the ICU and hospital of each patient was included as a secondary outcome.

### 2.5. Statistical Analysis

Data were analyzed and displayed based on types of variables and their distributions. For categorical variables, they are presented as numbers (percentages), which were tested using Fisher’s exact or Chi-square tests. For continuous variables, they are displayed as the median (25 to 75 percentiles) or mean ± standard deviation, which were tested using student’s *t*-test or Wilcoxon rank-sum tests.

Multivariate modeling of the relationship between vitamin D supplementation and in-hospital mortality was explored using a logistic regression model, and a Cox regression model was employed to reveal the relationship between vitamin D supplementation and 28-day or 90-day mortality. All potential confounders were analyzed in the multivariate regression models. The effect of vitamin D supplementation was presented as odds ratio (ORs) with a 95% confidence interval (CI) for in-hospital mortality and hazard ratio (HRs) with a 95% CI for 28-day and 90-day mortality. 

Propensity score matching (PSM) is a widely used method to balance the potential confounders in a study population and evaluate the robustness of the results. A 1:1 matching PSM analysis with no replacement was performed via the nearest neighbor. The caliper width for PSM analysis was 0.02 in the present study. All potential confounders were taken into consideration in the PSM cohort. A total of 303 patients in the control group were selected and 303 patients in the vitamin D supplementation group were selected based on Stata (version 14.0).

To minimize selection bias and balance the baseline characteristics, two approaches, inverse probability of treatment weighting (IPTW) and overlap weighting (OW), were used [28]. IPTW needs to calculate the propensity score (PS) and assigns a weight to each patient through PS, with the vitamin D supplementation group weight = 1/PS and the non-vitamin D supplementation group weight = 1/(1 − PS). After performing standardized PS weighting on each patient, a standard population was ultimately obtained. In the standard population, the bias between the vitamin D users and non-vitamin D users tended to be consistent, which means that the difference in efficacy between the two groups can be attributed to the vitamin D used. Compared with the simple 1:1 PSM, the IPTW method has the advantage of avoiding the loss of sample size. For OW, this is a PS method aimed at simulating important attributes of RCTs. OW assigns weights proportional to the probability of each patient belonging to the opposite treatment group. Briefly, patients who received vitamin D were weighted by the probability of not being supplemented with vitamin D (1 − PS). Patients who did not receive vitamin D were weighted by the probability of receiving vitamin D (PS). 

Then, subgroup analyses were performed in our study according to age, gender, septic shock, CHF, AKI, CKD, osteoporosis, and different doses of vitamin D supplementation. Survival analysis was conducted using Kaplan–Meier estimates, and we estimated the differences between the two survival curves using log-rank tests. 

For statistical analyses in this study, we used Stata (version 14.0), SPSS (version 23.0), and R language. A *p*-value < 0.05 was considered to indicate statistical significance.

## 3. Results

### 3.1. Baseline Characteristics and Grouping of Study Participants

**Figure 1:**
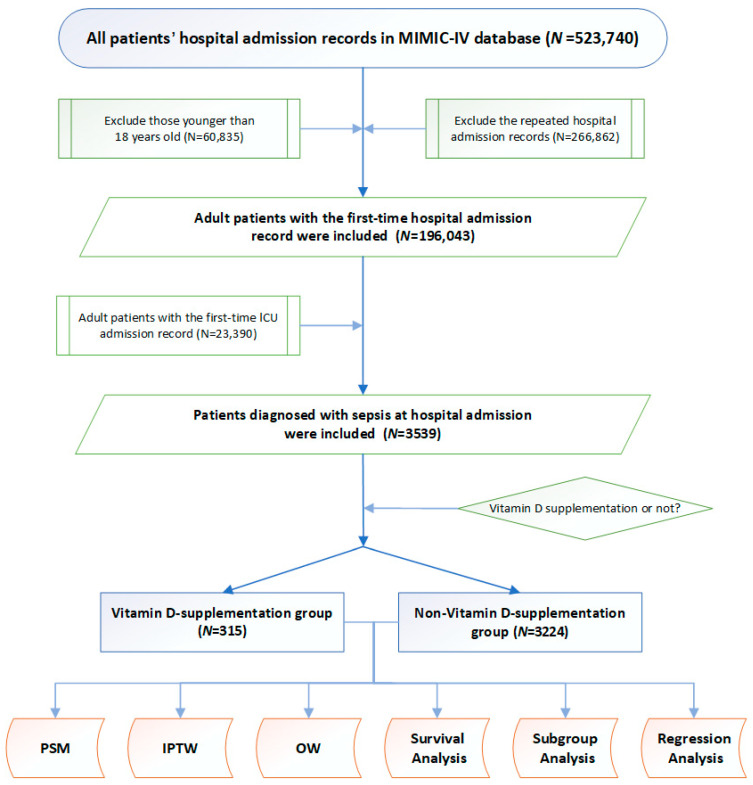
Study flowchart. MIMIC-IV database, the Mart for Intensive Care IV database; PSM, propensity score matching. ICU, intensive care unit. PSM, propensity score matching; IPTW, inverse probability of treatment weighting; OW, overlap weighting.

The study design is displayed as a flowchart in Figure 1. Based on the inclusion criteria, 3539 adult patients with sepsis were included as study participants. Among these, 315 patients were supplemented with vitamin D during their ICU stay, whereas 3224 patients were not. Specific indices, such as vital signs, comorbidities, clinical indices, and treatment measures, of each patient with sepsis were included for further analysis (Table 1). Compared with the non-vitamin D-medication group, patients supplemented with vitamin D were older (73 (60–84) vs. 68 (56–80)), had lower body weights (73 (62–91) vs. 79 (66–95)), and comprised a higher proportion of males (51.6% vs. 44.0%). Regarding comorbidities, the incidences of AF (8.0% vs. 5.3%), CHF (24.4% vs. 21.0%), and osteoporosis (16.2% vs. 4.9%) were significantly higher in the vitamin D supplementation group than in the non-supplementation group. Additionally, glucose (111 (88–136) vs. 119 (97–155)) and chloride (103 (100–108) vs. 104 (100–108)) levels were significantly lower in the patients with sepsis supplemented with vitamin D. No statistical differences were found in the other indices between the two groups. Notably, all patients in our study (3539 patients in total) were matched with the diagnosis of vitamin D deficiency based on the ICD-10 code (E559), but none of them were diagnosed with vitamin D deficiency at hospital admission.

### 3.2. Unadjusted Clinical Outcomes and Survival Analysis 

Table 2 displays all the observed primary outcomes, including in-hospital, 28-day, and 90-day mortality. Patients with sepsis supplemented with vitamin D faced a lower mortality risk in the hospital compared with those who were not supplemented with vitamin D (20.2% vs. 28.4%). Similarly, the vitamin D supplementation group had a lower 28-day (18.8% vs. 26.4%) and 90-day (23.5% vs. 29.9%) mortality rates, indicating that vitamin D supplementation during the ICU stay might be associated with improved short-term as well as long-term outcomes. 

Regarding secondary clinical outcomes, the non-vitamin D supplementation group showed significantly higher mean values of SOFA (7 (5–10) vs. 6 (4–8)), APS III (62 (47–83) vs. 55 (43–73)), and SAPI II (44 (35–52) vs. 42 (33–49)) scores than the vitamin D supplementation group during the ICU stay, indicating poorer health conditions of the patients in the non-vitamin D supplementation group. Notably, vitamin D supplementation was not associated with the LOS in the ICU and hospital. 

**Figure 2:**
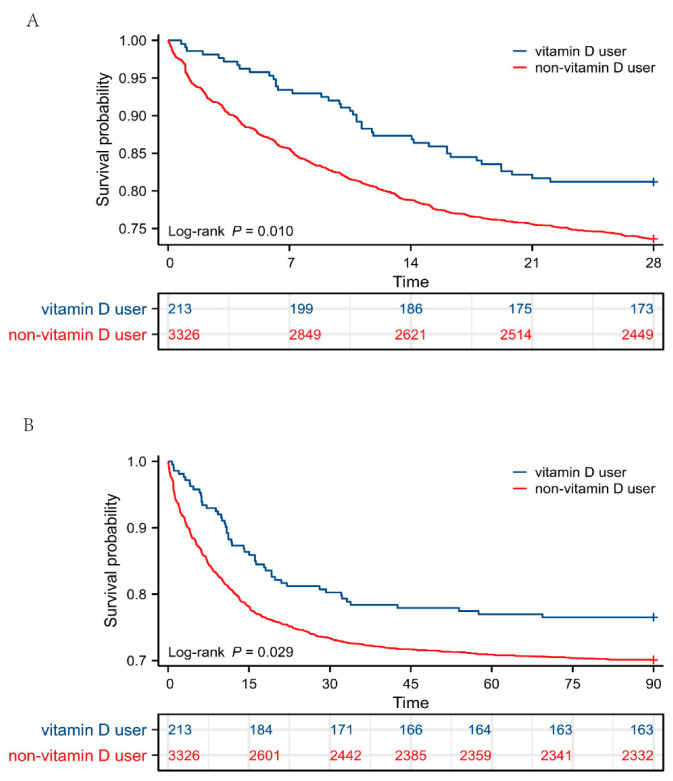
Survival analysis between vitamin D users and non-vitamin D users. (**A**) Kaplan–Meier survival curve of the two groups within 28 days and table of number at risk. (**B**) Kaplan–Meier survival curve of the two groups within 90 days and table of number at risk. The y axis shows the survival probability of patients over time, and the table of numbers at risk shows the number of survivors at each time point.

Additionally, Kaplan–Meier survival analysis was performed to intuitively reflect the survival probability of the two groups. As shown in Figure 2, patients with sepsis supplemented with vitamin D had significantly a higher probability of survival at 28 and 90 days (*p* < 0.05) than those not given vitamin D. Taken together, these data suggested that patients with sepsis might benefit from receiving vitamin D during their ICU stay to improve clinical outcomes. 

### 3.3. Association between Vitamin D Supplementation and Primary Outcomes Based on Multivariate Regression Analysis

Multivariate logistic regression analysis was performed to explore whether vitamin D supplementation could be a potential protective factor for patients with sepsis in reducing in-hospital mortality; multivariate Cox regression analysis was also performed for 28-day and 90-day mortality rates. As displayed in Table 3, in unadjusted model 1, the OR for the vitamin D supplementation group for in-hospital mortality was 0.68 (0.51–0.90), and the HRs for the vitamin D supplementation group for 28-day and 90-day mortality were 0.68 (0.52–0.88) and 0.75 (0.59–0.94), respectively. The relationships between each variable and mortality risk are displayed in Table S1.

After adjusting by age, gender, and weight in model 2, the results were relatively robust. The OR for in-hospital mortality in the vitamin D supplementation group was 0.64 (0.48–0.85), and the HRs for 28-day and 90-day mortality in the vitamin D supplementation group were 0.63 (0.49–0.82) and 0.70 (0.56–0.89), respectively. 

Next, model 3 was adjusted by age, gender, weight, presence of cerebral diseases, MI, CHF, CKD, AKI, diabetes, and osteoporosis, WBC, RBC, lactate, glucose, creatinine, platelet, and hemoglobin levels, septic shock, and use of antibiotics, MV, and vasopressin to eliminate the influence of all potential confounders and the trend remained consistent. The OR for the vitamin D supplementation group for in-hospital mortality was 0.69 (0.50–0.94), and the HRs for the vitamin D supplementation group for 28-day and 90-day mortality were 0.67 (0.48–0.92) and 0.74 (0.55–0.99), respectively. The results of multivariate regression analysis revealed that vitamin D supplementation during an ICU stay might play an important protective role in patients with sepsis. 

### 3.4. Baseline Characteristics, Clinical Outcomes Post PSM 

In the present study, we performed a 1:1 matched PSM analysis, and 606 patients were enrolled in the final cohort. Among them, 303 were supplemented with vitamin D and the remaining 303 were not. No significant differences were observed in the baseline characteristics between these two groups after PSM (Table 4). As displayed in Table 5, the primary outcomes showed that the vitamin D supplementation group had lower in-hospital mortality (21.5% vs. 29.7%), as well as 28-day (19.5% vs. 26.7%) and 90-day (23.8% vs. 32.3%) mortality rates. As for the disease severity-related scores, SOFA (6 (4–8) vs. 7 (4–10)) and APS III (54 (43–73) vs. 62 (48–82)) scores were significantly lower in the patients with sepsis supplemented with vitamin D compared to those who did not receive vitamin D. Although the SAPS II score (40 (32–49) vs. 42 (35–51)) was lower in the vitamin D supplementation group, no statistical differences were found between the two cohorts (*p* = 0.115). Consistently, there were no significant differences for the LOS in the ICU and hospital between the two groups (*p* = 0.988 and *p* = 0.207, respectively).

### 3.5. Regression Analysis and Survival Analysis Post PSM, IPTW, and OW

**Figure 3:**
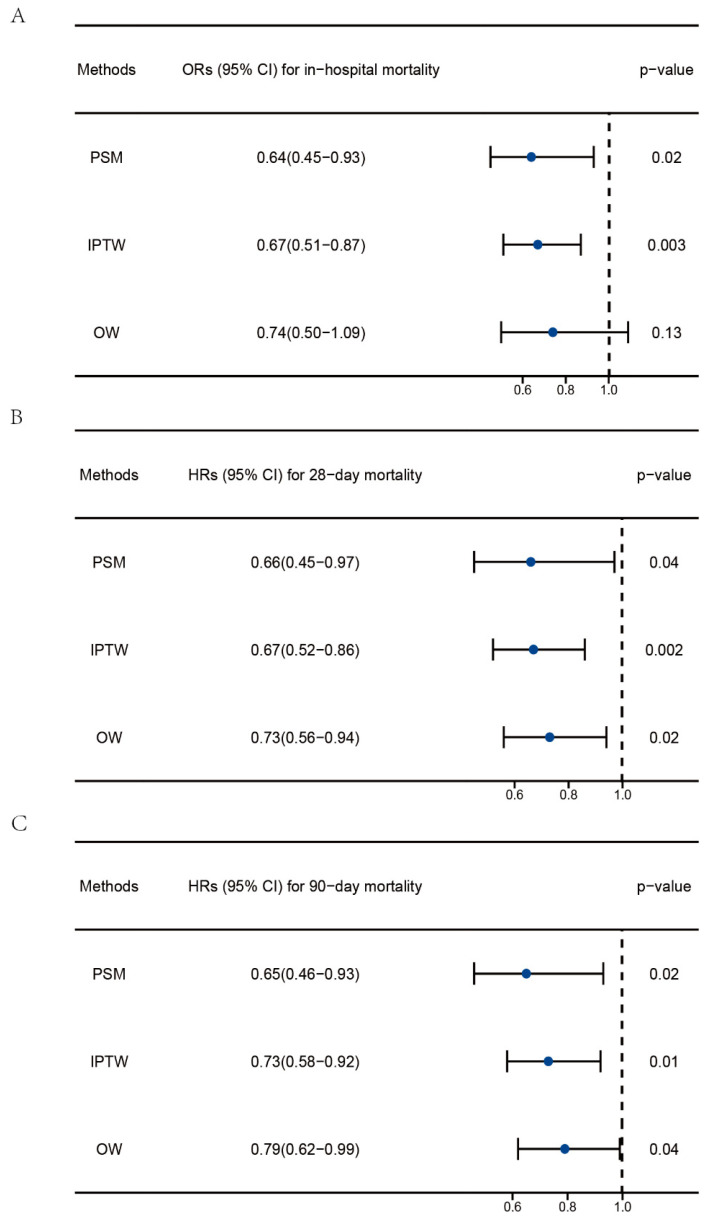
Regression analysis to explore the impact of vitamin D supplementation on the clinical outcomes of patients with sepsis post PSM, IPTW, and OW. (**A**) Association between vitamin D supplementation and in-hospital mortality. (**B**) Association between vitamin D supplementation and 28-day mortality. (**C**) Association between vitamin D supplementation and 90-day mortality. PSM, propensity score matching; IPTW, inverse probability of treatment weighting; OW, overlap weighting. The data are presented as the median (25 to 75 percentiles).

IPTW and OW were used to minimize selection bias and balance the baseline characteristics. The standardized mean differences (SMDs) of each variable post analysis are shown in Figure S1. After PSM and OW, the baseline demographic characteristics between the two groups were balanced with SMD < 0.1. Moreover, logistic regression and Cox regression were performed to explore the association between vitamin D supplementation and clinical outcomes post PSM, IPTW, and OW. As shown in Figure 3, the ORs of vitamin D supplementation were 0.64 (0.45–0.93) and 0.67 (0.51–0.87) for in-hospital mortality post PSM and IPTW, respectively. The OR for in-hospital mortality in the OW cohort was 0.74 (0.50–1.09) with a *p*-value of 0.13. The HRs of vitamin D supplementation for 28-day mortality were 0.66 (0.45–0.97), 0.67 (0.52–0.86), and 0.73 (0.56–0.94) post PSM, IPTW, and OW. The HRs of vitamin D supplementation for 90-day mortality were 0.65 (0.46–0.93), 0.73 (0.58–0.92), and 0.79 (0.62–0.99) post PSM, IPTW, and OW. 

**Figure 4:**
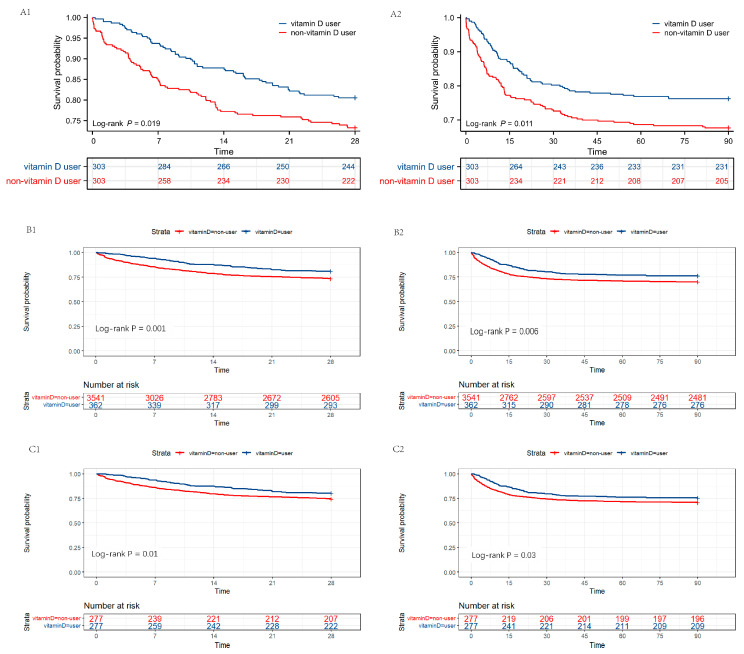
Survival analysis between vitamin D users and non-vitamin D users post PSM, IPTW, and OW. (**A1**,**A2**) Kaplan–Meier survival curves of the two groups within 28 days and 90 days post PSM. (**B1**,**B2**) Kaplan–Meier survival curves of the two groups within 28 days and 90 days post IPTW. (**C1**,**C2**) Kaplan–Meier survival curves of the two groups within 28 days and 90 days post OW. The y axis shows the survival probability of patients over time, and the table of numbers at risk shows the number of survivors at each time point. PSM, propensity score matching; IPTW, inverse probability of treatment weighting; OW, overlap weighting.

As displayed in Figure 4, the results of the survival analysis were consistent with previous results in the PSM-, IPTW- and OW-adjusted cohorts, validated by the log-rank test (*p* < 0.05). The Kaplan–Meier curves showed that patients treated with vitamin D had a higher probability of survival within 28 and 90 days post decline to a minimum selection bias. 

### 3.6. Subgroup Analysis

To confirm the protective effect of vitamin D supplementation in a specific population, multivariate regression analysis was performed to explore the association between vitamin D use and clinical outcomes in each subgroup (Table 6). Vitamin D supplementation was a potential protective factor in both male and female patients in reducing in-hospital, 28-day, and 90-day mortality rates, and the protective effect of vitamin D was independent of the presence of CHF, AKI, CKD, and osteoporosis. Additionally, the association between vitamin D supplementation and improved primary outcomes remained significant in patients older than 60 years and those with septic shock. Interestingly, the beneficial effects of vitamin D supplementation disappeared in younger patients (age < 60) and patients without septic shock.

**Figure 5:**
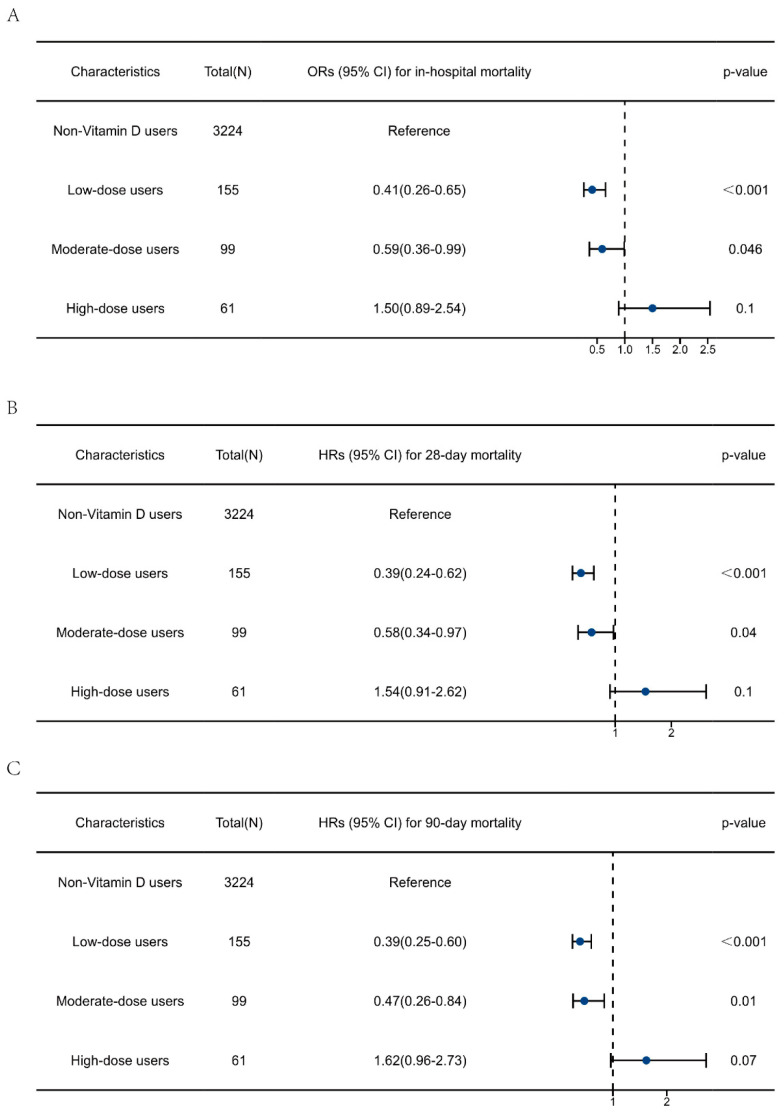
Multivariate regression analysis to explore the impact of vitamin D supplementation dosage on the clinical outcomes of patients with sepsis. (**A**) Association between vitamin D dosage and in-hospital mortality. (**B**) Association between vitamin D dosage and 28-day mortality. (**C**) Association between vitamin D dosage and 90-day mortality. The data are presented as the median (25 to 75 percentiles).

Additionally, the impact of different doses of vitamin D supplementation on outcomes was further explored. As displayed in Table S2, after adjusting for the dosage of vitamin D, the protective effects of vitamin D supplementation on sepsis outcomes remained robust. Multivariate regression analysis was then performed to explore the association between different doses of vitamin D supplementation and clinical outcomes (Figure 5). The results showed that both low-dose and moderate-dose vitamin D supplementation was associated with reduced risk of in-hospital, 28-day, and 90-day mortality in patients with sepsis when compared with non-vitamin D users. The ORs for in-hospital mortality were 0.41 (0.26–0.65) and 0.59 (0.36–0.99), respectively. The HRs for 28-day mortality were 0.39 (0.24–0.62) and 0.58 (0.34–0.97), and the HRs for 90-day mortality were 0.39 (0.25–0.60) and 0.47 (0.26–0.84), respectively. High-dose vitamin D supplementation showed no protective effect on sepsis outcomes. 

### 3.7. Post Hoc Analysis to Explore Whether Age and Severity of Sepsis Affect the Protective Effect of Vitamin D Supplementation

Age and septic shock were found to be independent risk factors for patients with sepsis (Table S1). As shown in Table 6, no interaction effects were found between vitamin D supplementation and age or septic shock. Baseline characteristics in younger patients (age < 60) and patients without septic shock (Tables S3 and S4) were further compared. In younger patients, the vitamin D users had a higher incidence of CKD (21.1% vs. 13.1%) and osteoporosis (15.8% vs. 2.0%), but lower proportions of antibiotic use (93.4% vs. 97.9%) and mechanical ventilation (72.4% vs. 81.2%). Patients without septic shock were older (74 (62–85) vs. 67 (55–80) years), comprised a higher proportion of males (52.4 vs. 43.1), and had greater weights (78 (65–93) vs. 72 (62–87)) in the vitamin D supplementation group, accompanied by a higher incidence of CHF (24.8% vs. 18.5%) and osteoporosis (15.9% vs. 4.5%). After PSM, no statistically significant differences were observed in the clinical outcomes of younger patients between the vitamin D supplementation group and the non-supplementation group (Table S5). However, in patients without septic shock, there was a trend of decreasing mortality risk and lower mean SOFA score (4.5 (3–7.6) vs. 5.5 (3.0–8.0)) in the vitamin D supplementation group (Table S6). 

## 4. Discussion

The debate on the benefits of vitamin D supplementation in critically ill populations continues, accompanied by controversial results. In this study, whether critically ill population with sepsis could benefit from vitamin D supplementation during their ICU stay to improve their prognosis was studied. The results showed that patients with sepsis treated with vitamin D had lower in-hospital, 28-day, and 90-day mortality rates as well as milder disease severity, indicated by lower SOFA and APS III scores. After PSM analysis, the improved outcomes remained robust. Multivariate regression analysis revealed that vitamin D supplementation might be a significant protective factor against in-hospital, 28-day, and 90-day mortality in patients with sepsis. Survival analysis also indicated that patients with sepsis supplemented with vitamin D had a higher survival probability. Taken together, our study suggests that vitamin D supplementation during an ICU stay might be associated with improved prognosis in critically ill patients with sepsis, which requires further multicenter and randomized controlled trials for validation. 

Sepsis, a life-threatening organ dysfunction caused by a dysregulated host response to infection, is characterized by an inadequate systemic immune response to an initial stimulus [29]. Until now, sepsis remains the primary cause of mortality in critically ill patients in the ICU, which is estimated at 31.5 million for patients with sepsis and 19.4 million for patients with severe sepsis worldwide with approximately 5.3 million deaths annually [30]. Up to now, there is no specific drug to treat sepsis. In addition to traditional strategies in clinical practice, such as infection control and hemodynamic management [31], nutrition support has been considered as an adjuvant therapy, with vitamin D supplementation as a promising option [32]. In the present study, patients in the vitamin D supplementation group were significantly older (73 (60–84) vs. 68 (56–80)) and had a higher incidence of osteoporosis (16.2% vs. 4.9%), which explains why they were given vitamin D.

Regarding the underlying mechanisms, the effects of vitamin D on infectious diseases may be related to its potential capacities as an immunomodulating agent [33,34]. In innate immunity, vitamin D combines with its receptor to increase the expression of antibacterial peptide LL-37 by activating the transcription of the antibacterial peptide gene [35]. Vitamin D reduces monocyte TLR expression while triggering hypo-responsiveness to bacterial cell wall components, thereby alleviating sepsis [36]. Concerning adaptive immunity, researchers have found that intravenous calcitriol administration post sepsis modulated the homeostasis of CD4+ T-cell populations associated with alleviating kidney injury induced by sepsis in obese mice [37]. Vitamin D also inhibits human B-cell activation stimulated by pokeweed mitogen [38]. Therefore, vitamin D plays an important protective role against sepsis by suppressing excessive immune responses to alleviate tissue damage. 

Additionally, vitamin D analogs might inhibit endotoxemia via regulation of free radicals and TXA2 formation, and improve the prognosis in LPS-treated mice [39]. Vitamin D has also been found to protect mice from oxidative damage by activating Nrf2-related signaling pathways and inhibiting the phosphorylation level of NF-κB [40]. Interestingly, the association between a high dose of vitamin D use and improved prognosis in patients with COVID-19 has been demonstrated via suppression of cytokine storms [41]. This evidence suggests that vitamin D might reduce the systemic level of inflammation and upregulate the antioxidant ability of patients with sepsis. Moreover, according to the Third International Consensus Definitions for Sepsis and Septic Shock (Sepsis-3), SOFA was recommended for screening sepsis and assessing prognoses [29], and showed an excellent ability for mortality discrimination [42]. Therefore, in our PSM cohort, we matched the SOFA score as well as APS III and SAPS II scores between two groups in the first 24 h during the ICU stay to avoid potential confounders. 

Age and septic shock have been identified as independent risk factors for bloodstream infections for more than 30 years [43]. In this study, the results showed that patients supplemented with vitamin D were significantly older than those not given vitamin D. Regarding potential reasons, as previously reported, elderly people are more likely to face the risk of vitamin D deficiency and osteoporosis [44,45]. Therefore, in clinical practice, doctors tend to use vitamin D supplements for elderly patients, which partly explains why patients who received vitamin D supplements were older. Furthermore, age and septic shock were independent risk factors for adverse clinical outcomes in patients with sepsis (Table S1) in our study. The prevalence of vitamin deficiency is higher in older patients than in younger adults [46], indicating that older patients might have more severe vitamin D deficiencies. Vitamin D deficiency is also prevalent in people with septic shock and is associated with increased mortality [47]. A randomized controlled trial conducted in India observed a trend toward a lower 90-day mortality rate in the severe vitamin D deficiency subgroup with serum levels lower than 12 ng/mL [25]. Similarly, an improvement in vitamin D status during the year leading up to hospitalization was independently associated with improved all-cause mortality rate and decreased hospital LOS in those with pre-hospital 25(OH)D concentrations < 20 ng/mL [48]. In our cohort, we analyzed patients with 25(OH)D values during the ICU stay and found that as serum 25(OH)D concentration increased, the mortality rate of patients gradually decreased (Figures S2 and S3). After balancing the 25(OH) D values between the two groups, a lower mortality rate was still observed in the vitamin D supplementation group, but there was no statistical difference due to population limitations (Table S7). Taken together, we hypothesized that the protective effect of vitamin D supplementation might be partly influenced by the patients’ serum vitamin D level. Although all patients in our cohort were not diagnosed with vitamin D deficiency at hospital admission, this situation might have occurred after entering the ICU, especially in older patients, which might explain why the beneficial effect of vitamin D supplementation disappeared in the younger population. This difference may be related to the lower proportion of vitamin D supplementation in younger patients and those without septic shock, which could impair the effectiveness of the statistical analysis.

There is still no consensus on whether vitamin D supplements should be given to critically ill population, especially those with sepsis, and how much or which route [11]. In this study, patients in the vitamin D supplementation group had a higher incidence of osteoporosis compared with those in the non-vitamin D supplementation group (16.2% vs. 4.9%). Vitamin D is considered the first step for treating patients with osteoporosis [12]. Hence, this may be one of the most important reasons why doctors choose to use vitamin D supplements for these patients. According to a clinical practice guideline published by the Endocrine Society in 2011, a daily requirement of 1500–2000 IU is recommended for patients at risk for vitamin D deficiency while the recommended dietary allowance of the Institute of Medicine (US) is 600 IU, and the tolerable upper intake level is 10,000 IU [27]. The doses of vitamin D supplementation in ICU patients varied from 200 to 540,000 IU in either single or repeated doses in previous studies with no clinically relevant adverse effects except for transient hypercalcemia [11,49]. Although a study showed that a single, oral, ultra-high dose of cholecalciferol corrects vitamin D deficiency rapidly in 80% of patients [50], it is still unclear whether such a relatively high dose will improve patient outcomes. In this study, we found that both low-dose and moderate-dose vitamin D supplementation (<2000 IU) had protective effects on the clinical outcomes of sepsis, whereas a high dose did not. In the high-dose group, the disappearance of the protective effects may be due to the limited number of patients, which limited the effectiveness of the statistical analysis. Taken together, more evidence is still needed to assist clinicians in determining the most appropriate dosage of vitamin D supplementation for patients with sepsis. Regarding the LOS in the hospital and ICU, a greater portion of critically ill patients in the non-vitamin D supplementation group died at an early stage on admission to the ICU and hospital, which might have shortened the overall LOS of this group.

A previous temporal trend study conducted in the United States (US) observed a decrease in in-hospital mortality among a population with severe sepsis from 2003 to 2007 [51]. Furthermore, from 2010 to 2015, the in-hospital mortality rate for sepsis hospitalizations declined from 24.1% to 14.8% [52]. Interestingly, the usage rate of vitamin D supplements and the serum 25(OH)D concentrations showed opposite temporal trends in the US population. Researchers have found that serum 25(OH)D showed modest increases during the period from 2007 to 2010 in a US population, possibly due to the surge in the use of vitamin supplements over the past decade [53]. In a multicenter cohort, women whose 25(OH)D measured in 2009–2011 was 16 nmol/L higher than that measured in 1998–2000 after adjusting for confounders, and the increase in 25(OH)D was greater in vitamin D supplement users [54]. Similar results showed that the overall utilization of vitamin D supplements has increased between 2000 and 2009 in the US, especially in females, elderly people, and patients with osteoporosis [55]. Taken together, these evidence indicate that over the past few decades, the hospital mortality rate of sepsis patients has gradually decreased, accompanied by increased use of vitamin supplements and higher serum 25(OH)D levels among the US population, suggesting a potential correlation between them. However, the improvement in the prognosis of patients with sepsis in the past few decades may also be related to some other factors, such as policies [56] and improved treatment strategies [57,58]. Therefore, the causal relationship between sepsis outcomes and vitamin D supplementation requires further research.

Indeed, sepsis is a syndrome encompassing a still uncertain pathobiology instead of a specific illness with no gold standard diagnostic tests [29]. Significant changes in sepsis definitions and coding criteria over time may have non-negligible impacts on the incidence of sepsis and sepsis-associated mortality [56]. Additionally, compared with sepsis-1, the sepsis-3 guidelines narrowed the sepsis population at the expense of sensitivity, which may result in false negatives [59]. In this study, although MIMIC-IV database identified patients with sepsis based on the latest sepsis-3 guidelines and ICD-9 code, the effects of a long time span (2008–2019) on sepsis incidence and outcomes may still exist. Thus, this limits the direct comparison of results between different studies. 

The selection bias in the cohort study might affect the reliability of the results [28]. Inverse probability of treatment weighting (IPTW) and OW are widely adopted approaches to handle selection bias and account for confounders [60,61,62]. We found that OW had a better performance at balancing baselines to minimize selection bias compared to IPTW in our study participants (Figure S1). In the IPTW cohort, vitamin D supplementation was associated with lower in-hospital, 28-day and 90-day mortality risk, and vitamin D supplementation was associated with lower 28-day and 90-day mortality risk post decline to a minimum selection bias using OW in patients with sepsis. Hence, by using IPTW and OW analyses, we further validated the results in our study participants. 

To the best of our knowledge, this is the first retrospective study to explore the association between vitamin D supplementation and prognosis in patients with sepsis based on high-quality data from a large critically ill cohort. Moreover, several statistical analyses were adopted to minimize selection bias and balance the baseline characteristics. However, there are still some limitations to the present study. Firstly, this is a retrospective study conducted in a single center, and although we have adopted various methods to exclude potential confounders, they may still exist. Secondly, there might be some unmeasured confounders that affect the pharmacodynamics of vitamin D, such as a history of gastrointestinal surgery, nutrition supply during hospitalization, and accompanying with liver failure, which are noteworthy for further study. Moreover, we did not explore the effect of the specific time of vitamin D supplementation on the prognosis of patients with sepsis. Therefore, future multicenter and large-scale RCTs with more representative populations are required to verify our results.

## 5. Conclusions

Vitamin D supplementation during the ICU stay is associated with improved prognosis in patients with sepsis, as evidenced by lower in-hospital, 28-day, and 90-day mortality rates with no influence on the LOS in the hospital and ICU. 

## Figures and Tables

**Table 1:** Baseline characteristics of study participants.

	All Septic Patients (*n* = 3539)	Non-Vitamin D Supplementation Group (*n* = 3224)	Vitamin D Supplementation Group (*n* = 315)	*p*-Value
**Demographic data**				
Age (years)	68 (56–80)	68 (56–80)	73 (60–84)	<0.001
Male (*n* (%))	1573 (44.4)	1463 (44.0)	110 (51.6)	0.01
Weight (kg)	78 (65–95)	79 (66–95)	73 (62–91)	0.03
**Vital signs**				
RR (/min)	21 (18–24)	21 (18–24)	21 (18–24)	0.06
SBP (mmHg)	113 (98–130)	113 (98–130)	111 (97–130)	0.08
DBP (mmHg)	57 (49–67)	57 (49–67)	59 (49–69)	0.59
Temperature (°C)	36.9 (36.6–37.3)	36.9 (36.6–37.3)	36.8 (36.6–37.2)	0.35
**Comorbidities**				
Cerebral diseases (*n* (%))	399 (11.3)	356 (11.0)	43 (13.7)	0.16
MI (*n* (%))	303 (8.6)	279 (8.7)	24 (7.6)	0.60
AF (*n* (%))	192 (5.4)	175 (5.3)	17 (8.0)	0.049
CHF (*n* (%))	749 (21.2)	697 (21.0)	52 (24.4)	0.001
CKD (*n* (%))	858 (24.2)	804 (24.2)	54 (25.4)	0.30
AKI (*n* (%))	2257 (63.8)	2132 (64.1)	125 (58.7)	0.36
Diabetes (*n* (%))	1171 (33.1)	1054 (32.7)	117 (37.1)	0.12
Osteoporosis (*n* (%))	208 (5.9)	157 (4.9)	51 (16.2)	<0.001
Septic shock (*n* (%))	2043 (57.7)	1930 (58.0)	113 (53.1)	0.17
**Clinical indices**				
RBC (m/μL)	3.3 (2.9–3.8)	3.3 (2.9–3.8)	3.3 (2.9–3.8)	0.61
WBC (K/μL)	10.3 (6.9–15.5)	10.4 (7.0–15.5)	9.2 (6.0–14.7)	0.75
Platelet (K/μL)	191 (123–282)	190 (123–282)	203 (127–284)	0.30
Hemoglobin (g/dL)	9.9 (8.7–11.2)	9.9 (8.7–11.3)	9.8 (8.5–11.2)	0.21
Creatinine (mg/dL)	1.1 (0.7–2.0)	1.1 (0.7–2.0)	1.1 (0.7–1.9)	0.21
Glucose (mmol/L)	118 (96–154)	119 (97–155)	111 (88–136)	0.002
Lactate (mg/dL)	1.7 (1.2–2.8)	1.7 (1.2–2.8)	1.7 (1.2–2.9)	0.30
Potassium (mmol/L)	4.0 (3.7–4.4)	4.0 (3.7–4.4)	4.0 (3.7–4.4)	>0.90
Chloride (mmol/L)	104 (100–108)	104 (100–108)	103 (100–108)	0.03
**Treatment measures**				
Vasopressin (*n* (%))	837 (23.7)	784 (23.6)	53 (24.9)	0.63
Antibiotic (*n* (%))	3468 (98.0)	3262 (81.1)	206 (96.7)	0.29
MV (*n* (%))	2991 (84.5)	2816 (84.7)	175 (82.2)	0.33

RR, respiratory rate; SBP, systolic blood pressure; DBP, diastolic blood pressure; MI, myocardial infarction; AF, atrial fibrillation; CHF, chronic heart failure; CKD, chronic kidney disease; AKI, acute kidney injury; RBC, red blood cell; WBC, white blood cell; MV, mechanical ventilation. Categorical variables are presented as numbers (percentages) and continuous variables are presented as the median (25 to 75 percentiles).

**Table 2:** Clinical outcomes of study participants.

Clinical Outcomes	All Septic Patients (*n* = 3539)	Non-Vitamin D Supplementation Group (*n* = 3224)	Vitamin D Supplementation Group (*n* = 315)	*p*-Value
**Primary outcomes**				
In-hospital mortality (*n* (%))	987 (27.9)	944 (28.4)	43 (20.2)	0.01 *
28-mortality (*n* (%))	917 (25.9)	877 (26.4)	40 (18.8)	0.01 *
90-mortality (*n* (%))	1044 (29.5)	994 (29.9)	50 (23.5)	0.03 *
**Secondary outcomes**				
Mean SAPS II	44 (35–52)	44 (35–52)	42 (33–49)	0.04 *
Mean APS III	62 (46–82)	62 (47–83)	55 (43–73)	<0.001 *
Mean SOFA	7 (4–10)	7 (5–10)	6 (4–8)	<0.001 *
ICU LOS (days)	3.0 (1.6–7.2)	3.0 (1.7–7.3)	2.7 (1.6–5.6)	0.12
Hospital LOS (days)	9.9 (5.5–17.9)	9.9 (5.5–18.0)	9.7 (5.7–16.2)	0.11

SAPS II, Simplified Acute Physiology Score-II; APS III, Acute Physiology Score III; SOFA, Sequential Organ Failure Assessment; LOS, lengths of stay. Categorical variables are presented as numbers (percentages) and continuous variables are presented as the median (25 to 75 percentiles). * *p*-value < 0.05.

**Table 3:** Association between vitamin D supplementation and clinical outcomes using multivariate regression analysis.

	HRs for 28-Day Mortality	HRs for 90-Day Mortality	ORs for in-Hospital Mortality
Model 1	0.68 (0.52–0.88)	0.75 (0.59–0.94)	0.68 (0.51–0.90)
Model 2	0.63 (0.49–0.82)	0.70 (0.56–0.89)	0.64 (0.48–0.85)
Model 3	0.67 (0.48–0.92)	0.74 (0.55–0.99)	0.69 (0.50–0.94)

Model 1 was unadjusted. Model 2 was adjusted by age, gender, and weight. Model 3 was adjusted by age, gender, weight, presence of cerebral disease, myocardial infarction, chronic kidney disease, acute kidney injury, diabetes, and osteoporosis, white blood cell, red blood cell, lactate, glucose, creatinine, platelet, hemoglobin, potassium, and chloride levels, septic shock, and use of antibiotics, mechanical ventilation, and vasopressin. The data are presented as the median (25 to 75 percentiles).

**Table 4:** Baseline characteristics of study participants after propensity score matching.

	All Septic Patients (*n* = 606)	Non-Vitamin D Supplementation Group (*n* = 303)	Vitamin D Supplementation Group (*n* = 303)	*p*-Value
**Demographic data**				
Age (years)	72 (60–83)	72 (61–82)	72 (60–85)	0.42
Male (*n* (%))	317 (52.3)	158 (52.1)	159 (52.5)	>0.90
Weight (kg)	76 (64–92)	77 (65–92)	76 (64–91)	0.57
**Vital signs**				
RR (/min)	21 (18–24)	21 (18–24)	21 (18–23)	0.33
SBP (mmHg)	111 (96–129)	111 (96–127)	111 (96–130)	0.74
DBP (mmHg)	57 (48–67)	55 (45–66)	59 (50–69)	0.08
Temperature (°C)	36.8 (36.6–37.3)	36.9 (36.6–37.3)	36.8 (36.6–37.2)	0.22
**Comorbidities**				
MI (*n* (%))	48 (7.9)	25 (8.3)	23 (7.6)	0.89
AF (*n* (%))	46 (7.6)	23 (7.6)	23 (7.6)	>0.90
HF (*n* (%))	164 (27.1)	78 (25.7)	86 (28.4)	0.52
CKD (*n* (%))	171 (28.2)	92 (30.4)	79 (26.1)	0.28
AKI (*n* (%))	379 (62.5)	195 (64.4)	184 (60.7)	0.40
Diabetes (*n* (%))	239 (39.4)	128 (42.2)	111 (36.6)	0.18
Osteoporosis (*n* (%))	106 (17.5)	56 (18.5)	50 (16.5)	0.60
Septic shock (*n* (%))	330 (54.5)	166 (54.8)	164 (54.1)	>0.90
Cerebral diseases (*n* (%))	75 (12.4)	37 (12.2)	38 (12.5)	>0.90
**Clinical indices**				
RBC (m/μL)	3.3 (2.9–3.8)	3.3 (2.9–3.9)	3.3 (2.9–3.7)	0.44
WBC (K/μL)	9.7 (6.2–15.3)	9.6 (6.2–15.3)	9.7 (6.1–15.2)	0.60
Platelet (K/μL)	191 (129–272)	187 (129–260)	201 (129–283)	0.44
Hemoglobin (g/dL)	9.9 (8.6–11.2)	9.9 (8.7–11.2)	9.9 (8.6–11.2)	0.40
Creatinine (mg/dL)	1.1 (0.7–1.8)	1.1 (0.8–1.8)	1.0 (0.7–1.7)	0.41
Glucose (mmol/L)	113 (92–142)	112 (94–145)	113 (90–142)	0.66
Lactate (mg/dL)	1.7 (1.2–2.8)	1.7 (1.1–2.9)	1.7 (1.2–2.8)	0.83
Potassium (mmol/L)	4.1 (3.7–4.5)	4.0 (3.7–4.5)	4.1 (3.7–4.4)	0.56
Chloride (mmol/L)	103 (100–108)	104 (100–108)	103 (100–107)	0.59
**Severity-related scores**				
SOFA	6 (4–9)	6 (4–9)	6 (4–9)	>0.90
SAPS II	42 (35–50)	42 (36–50)	41 (34–50)	>0.90
APS III	61 (47–75)	61 (49–78)	61 (46–72)	0.09
**Clinical measures**				
Vasopressin (*n* (%))	167 (27.6)	88 (29.0)	79 (26.1)	0.47
Antibiotic (*n* (%))	588 (97.0)	294 (97.0)	294 (97.0)	>0.90
MV (*n* (%))	501 (82.7)	251 (82.8)	250 (82.5)	>0.90

RR, respiratory rate; SBP, systolic blood pressure; DBP, diastolic blood pressure; MI, myocardial infarction; AF, atrial fibrillation; CHF, chronic heart failure; CKD, chronic kidney disease; AKI, acute kidney injury; RBC, red blood cell; WBC, white blood cell; MV, mechanical ventilation. Categorical variables are presented as numbers (percentages) and continuous variables are presented as the median (25 to 75 percentiles).

**Table 5:** Clinical outcomes after propensity score matching between vitamin D supplementation group and non-vitamin D supplementation group.

Clinical Outcomes	All Septic Patients (*n* = 606)	Non-Vitamin D Supplementation Group (*n* = 303)	Vitamin D Supplementation Group (*n* = 303)	*p*-Value
**Primary outcomes**				
In-hospital mortality (*n* (%))	155 (25.6)	90 (29.7)	65 (21.5)	0.03 *
28-day mortality (*n* (%))	140 (23.1)	81 (26.7)	59 (19.5)	0.04 *
90-day mortality (*n* (%))	170 (28.1)	98 (32.3)	72 (23.8)	0.02 *
**Secondary outcomes**				
Mean SAPS II	41 (34–50)	42 (35–51)	40 (32–49)	0.12
Mean APS III	59 (45–78)	62 (48–82)	54 (43–73)	0.001 *
Mean SOFA	6 (4–9)	7 (4–10)	6 (4–8)	<0.001 *
ICU LOS (days)	2.8 (1.6–6)	3 (1.6–6.6)	2.7 (1.5–5.6)	>0.90
Hospital LOS (days)	8.9 (5.4–15.7)	8.3 (5.0–15.0)	9.7 (5.7–16.4)	0.21

SAPS II, Simplified Acute Physiology Score-II; APS III, Acute Physiology Score III; SOFA, Sequential Organ Failure Assessment; LOS, length of stay. Categorical variables are presented as numbers (percentages) and continuous variables are presented as the median (25 to 75 percentiles). * *p*-value < 0.05.

**Table 6:** Association between vitamin D treatment and clinical outcomes stratified by age, gender, and presence of septic shock, CHF, AKI, CKD, and osteoporosis.

	Number of Patients	OR for in-Hospital Mortality	HR for 28-Day Mortality	HR for 90-Day Mortality	*p*-Values for Interaction
**Age**					0.08
>60	2421	0.57 (0.42–0.79)	0.62 (0.46–0.82)	0.67 (0.49–0.93)	
<60	1118	1.01 (0.58–1.76)	0.85 (0.46–1.57)	1.10 (0.64–1.88)	
**Gender**					0.75
Male	1573	0.65 (0.44–0.96)	0.63 (0.44–0.90)	0.71 (0.52–0.98)	
Female	1966	0.63 (0.42–0.96)	0.63 (0.43–0.93)	0.68 (0.48–0.97)	
**Septic shock**					0.10
Yes	2043	0.60 (0.41–0.86)	0.62 (0.45–0.86)	0.67 (0.47–0.86)	
No	1496	0.88 (0.56–1.37)	0.84 (0.53–1.34)	1.06 (0.70–1.60)	
**CHF**					0.53
Yes	749	0.56 (0.31–0.99)	0.56 (0.33–0.95)	0.60 (0.37–0.98)	
No	2790	0.70 (0.50–0.91)	0.67 (0.50–0.91)	0.75 (0.58–0.99)	
**AKI**					0.80
Yes	2257	0.70 (0.50–0.98)	0.68 (0.50–0.92)	0.76 (0.58–0.99)	
No	1282	0.55 (0.32–0.95)	0.58 (0.35–0.96)	0.62 (0.39–0.99)	
**CKD**					0.53
Yes	858	0.56 (0.32–0.99)	0.57 (0.34–0.96)	0.62 (0.38–0.99)	
No	2681	0.71 (0.51–0.99)	0.67 (0.50–0.91)	0.72 (0.55–0.95)	
**osteoporosis**					0.75
Yes	208	0.35 (0.15–0.79)	0.37 (0.17–0.82)	0.42 (0.21–0.85)	
No	3331	0.69 (0.51–0.94)	0.68 (0.52–0.90)	0.75 (0.59–0.97)	

CHF, chronic heart failure; CKD, chronic kidney disease; AKI, acute kidney injury. The data are presented as the median (25 to 75 percentiles).

## Data Availability

All the data are contained within the article.

## Supplementary Materials

The following supporting information can be downloaded at: https://www.mdpi.com/article/10.3390/nu15132924/s1, Table S1: ORs and HRs of each factor in adjusted model 3; Table S2: Association between Vitamin D supplementation and clinical outcomes using multivariate regression analysis after adjusting for doses of Vitamin D; Table S3: The baseline information and outcome of young patients with sepsis (age < 60 years); Table S4: The baseline characteristics of septic patients without septic shock; Table S5: Clinical outcomes of young patients with sepsis (age < 60 years) after PSM; Table S6: Clinical outcomes of septic patients without septic shock after PSM; Table S7: Clinical outcomes in patients with serum 25(OH)D values before and after PSM; Figure S1: Standardized mean differences of baseline characteristics in unmatched, 1:1 PSM, IPTW and OW-adjusted cohort. PSM, propensity score matching; IPTW, inverse probability of treatment weighting; OW, overlap weighting. Figure S2: Flow chart of identifying patients with serum 25(OH)D values. Figure S3: Association between serum 25(OH)D and in-hospital mortality in patients with serum 25(OH)D values. The y axis represents the survival probabilities of patients.
