# Supplementary material for: Vitamin D Supplementation during Intensive Care Unit Stay Is Associated with Improved Outcomes in Critically Ill Patients with Sepsis: A Cohort Study
Source: Nutrients. 2023 Jun 28;15(13):2924. doi: 10.3390/nu15132924 (PMC10346786; doi:10.3390/nu15132924)
Supplement: Supplementary file 1 [file nutrients-15-02924-s001.zip › nutrients-2433713-supplementary.pdf]

Vitamin D Supplementation during Intensive Care Unit Stay IsAssociated with Improved Outcomes in Critically Ill Patientswith Sepsis: A Cohort Study

Supplemental Materials

Table S1 ORs and HRs of each factor in adjusted model 3

Adjusted factors	ORs for in-hospital mortality	HRs for 28-day mortality	HRs for 90-day mortality
Age (years)	1.02(1.01-1.02)	1.02(1.01-1.02)	1.01(1.01-1.02)
Male (n (%))	\	\	\
Weight (Kg)	\	0.996(0.993-0.999)	\
RR (/min)	1.05(1.03-1.07)	1.05(1.04-1.07)	1.05(1.03-1.06)
SBP (mmHg)	0.99(0.986-0.994)	0.991(0.987-0.994)	0.991(0.988-0.994)
Temperature (℃)	0.61(0.53-0.70)	0.69(0.62-0.76)	0.68(0.62-0.74)
MI (n (%))	\	\	\
CHF (n (%))	0.65(0.51-0.81)	0.75(0.63-0.89)	0.73(0.62-0.97)
CKD (n (%))	\	\	\
AKI (n (%))	\	\	\
Diabetes (n (%))	\	\	\
Osteoporosis (n (%))	\	\	\
Septic shock (n (%))	1.62(1.35-1.94)	1.54(1.33-1.78)	1.50(1.31-1.72)
Cerebral diseases (n (%))	\	\	\
RBC (m/uL)	0.72(0.63-0.82)	\	0.86(0.78-0.95)
WBC (K/uL)	1.04(1.03-1.05)	1.01(1.01-1.02)	1.01(1.01-1.02)
Platelet (K/uL)	0.999(0.998-0.999)	0.999(0.988-0.994)	0.999(0.998-0.999)
Hemoglobin (g/dL)	\	\	\
Potassium (mmol/L)	1.36(1.20-1.55)	1.35(1.23-1.47)	1.32(1.21-1.44)
Chloride (mmol/L)	\	\	\
Creatinine(mg/dL)	1.16(1.09-1.23)	1.14(1.10-1.18)	1.11(1.08-1.15)
Glucose (mmol/L)	1.004(1.002-1.005)	1.002(1.001-1.003)	1.002(1.001-1.003)
Lactate (mg/dL)	1.29(1.23-1.35)	1.19(1.17-1.22)	1.19(1.17-1.21)
Vasopressin (n (%))	\	\	\
Antibiotic (n (%))	\	\	\
MV (n (%))	2.41(1.80-3.24)	2.09(1.63-2.69)	1.91(1.52-2.39)

RR, respiratory rate; SBP, systolic blood pressure; MI, myocardial infarction; CHF, chronic heart failure; CKD, chronic kidney disease; AKI, acute kidney injury; RBC, red blood cell; WBC, white blood cell; MV, mechanical ventilation.

Table S2 Association between Vitamin D supplementation and clinical outcomes using multivariate regression analysis after adjusting for doses of Vitamin D

Vitamin D Supplementation	Number of patients	ORs for in-hospital mortality	HRs for 28-day mortality	HRs for 90-day mortality
No	3224	Ref	Ref	Ref
Yes	315	0.69(0.52-0.91)	0.66(0.50-0.89)	0.75(0.57-0.99)

Ref, reference group.

Table S3 The baseline information and outcome of young patients with sepsis (age < 60 years)

	Before PSM			After PSM		
	Non-VD supplementation group (n= 3224)	VD-supplementation group (n= 315)	p-value	Non-VD supplementation group (n= 70)	VD-supplementation group (n= 70)	p-value
**Demographic data**						
Age (years）	51(42-56)	52(44-56)	0.22	52(43-56)	52(43-56)	0.78
Male (n (%))	457(43.9)	35(46.1)	0.40	24(34.3)	31(44.3)	0.15
Weight (Kg)	80(68-100)	84(68-107)	0.58	88(69-102)	84(69-107)	0.51
**Vital sign**s						
RR (/min)	22(18-25)	21(17.1-25)	0.37	21(18-25)	21(17-25)	0.73
SBP (mmHg)	109(96-126)	108(95-123)	0.56	117(99-127)	108(93-124)	0.27
DBP (mmHg)	61(52-70)	62(52-76)	0.46	63(52-75)	64(52-76)	0.67
Temperature (°C)	37.1(36.7-37.6)	37.0(36.7-37.6)	0.83	37.1(36.8-37.7)	37.1(36.7-37.6)	0.54
**Comorbidities**						
MI (n (%))	54(5.2)	1(1.3)	0.10	3(4.3)	1(1.4)	0.31
AF (n (%))	44(4.2)	7(9.2)	0.052	11(15.7)	6(8.6)	0.15
CHF (n (%))	82(7.9)	10(13.2)	0.09	13(18.6)	10(14.3)	0.32
CKD (n (%))	136(13.1)	16(21.1)	0.04	10(14.3)	15(21.4)	0.19
AKI (n (%))	581(55.8)	42(55.3)	0.51	36(51.4)	37(52.9)	0.50
Diabetes (n (%))	274(26.3)	26(34.2)	0.09	19(27.1)	24(34.3)	0.23
Osteoporosis (n (%))	21(2.0)	12(15.8)	<0.001	13(18.6)	10(14.3)	0.32
Cerebral diseases (n (%))	113(10.8)	6(7.9)	0.28	5(7.1)	6(8.6)	0.50
**Clinical indices**						
RBC (m/uL)	3.3(2.8-3.8)	3.2(2.8-3.9)	0.72	3.4(2.8-3.9)	3.2(2.8-4.0)	0.81
WBC (K/uL)	10.2(6.6-15.3)	8.0(5.9-14.0)	0.18	10.1(5.8-15.2)	8.0(5.8-15.3)	0.80
Platelet (K/uL)	178(112-272)	177(112-285)	0.78	178(121-253)	177(113-276)	>0.90
Hemoglobin	9.8(8.5-11.3)	10.0(8.4-11.7)	0.83	9.5(8.7-11.3)	10.0(8.4-11.7)	0.49
Creatinine(mg/dL)	1.0(0.6-1.7)	1.1(0.6-1.9)	0.61	1.0(0.6-2.0)	1.0(0.6-2.0)	0.73
Glucose (mmol/L)	115(95-147)	113(91-145)	>0.90	115(92.6-142.8)	108(90.8-137.5)	0.60
Lactate	1.7(1.1-2.9)	1.6(1.1-2.6)	0.56	1.6(1.0-3.0)	1.6(1.2-2.8)	>0.90
Potassium	4.0(3.6-4.4)	4.0(3.7-4.3)	0.54	4.10(3.7-4.4)	4.0(3.8-4.3)	0.34
Chloride	103(99-108.0)	103.0(98-107)	0.33	101(98-105)	103(98-107)	0.53
**Clinical measures**						
Vasopressin (n (%))	243(23.3)	21(27.6)	0.22	16(22.9)	19(27.1)	0.35
Antibiotic (n (%))	1020(97.9)	71(93.4)	0.03	66(94.3)	65(92.9)	0.50
MV (n (%))	846(81.2)	55(72.4)	0.046	55(78.6)	52(74.3)	0.35

PSM, propensity score matching; RR, respiratory rate; SBP, systolic blood pressure; DBP, diastolic blood pressure; MI, myocardial infarction; AF, atrial fibrillation; CHF, chronic heart failure; CKD, chronic kidney disease; AKI, acute kidney injury; RBC, red blood cell; WBC, white blood cell; MV, mechanical ventilation.

Table S4 The baseline characteristics of septic patients without septic shock

	Before PSM			After PSM		
	Non-VD supplementation group (n= 3224)	VD-supplementation group (n= 315)	*p*-value	Non-VD supplementation group (n= 138)	VD-supplementation group (n= 138)	*p*-value
**Demographic data**						
Age (years）	67(55-80)	74(62-85)	0.03	71(60-84)	73(62-86)	0.52
Male (n (%))	582(43.1)	76(52.4)	0.02	69(50.0)	70(50.7)	0.50
Weight (Kg)	78(65-93)	72(62-87)	0.02	76(63-92)	72(62-71)	0.36
**Vital signs**						
RR (/min)	21(18-24)	21(18-24)	0.46	20(18-23)	21(18-24)	0.46
SBP (mmHg)	116(101-134)	115(100-136)	0.49	115(101-134)	116(100-137)	0.90
DBP (mmHg)	59(50-69)	59(49-70)	0.83	58(49-65)	60(49-71)	0.16
Temperature (℃)	36.9(36.6-37.4)	36.9(36.6-37.2)	0.15	36.9(36.5-37.3)	36.9(36.6-37.2)	>0.90
**Comorbidities**						
MI (n (%))	116(8.6)	12(8.3)	0.53	11(8.0)	12(8.7)	0.50
AF (n (%))	58(4.3)	9(6.2)	0.19	11(8.0)	9(6.5)	0.41
CHF (n (%))	250(18.5)	36(24.8)	0.045	41(29.7)	36(29.1)	0.30
CKD (n (%))	293(21.7)	37(25.5)	0.17	39(28.3)	36(26.1)	0.39
AKI (n (%))	759(56.2)	79(54.5)	0.38	78(56.5)	77(55.58)	0.50
Diabetes (n (%))	436(32.3)	48(33.1)	0.45	51(37.0)	47(34.1)	0.35
Osteoporosis (n (%))	61(4.5)	23(15.9)	<0.001	20(14.5)	21(15.2)	0.50
Cerebral diseases (n (%))	145(10.7)	18(12.4)	0.31	19(13.8)	18(13.0)	0.50
**Cinical indices**						
RBC (m/uL)	3.4(2.9-3.8)	3.3(2.9-3.7)	0.72	3.4(2.9-3.9)	3.3(2.9-3.7)	0.68
WBC (K/uL)	9.7(6.6-14.3)	10.2(7.1-14.6)	0.77	10.7(7.0-14.2)	10.2(7.0-14.5)	>0.90
Platelet (K/uL)	195(125-288)	204(138-259)	0.37	198(142-289)	205(142-289)	0.87
Hemoglobin	10.1(8.8-11.4)	9.9(8.7-11.5)	0.46	10.2(8.8-11.5)	10.0(8.7-11.5)	0.70
Creatinine(mg/dL)	1.0(0.7-1.7)	1.0(0.7-1.7)	>0.90	1.1(0.7-1.7)	1.0(0.7-1.6)	0.77
Glucose (mmol/L)	118(97-151)	108(88-144)	0.09	107(92-134)	109(88-144)	0.31
Lactate	1.6(1.1-2.5)	1.7(1.2-2.5)	0.65	1.7(1.2-2.6)	1.8(1.2-2.6)	0.69
Potassium	4.0(3.7-4.4)	4.1(3.8-4.5)	0.33	4.0(3.7-4.4)	4.1(3.8-4.5)	0.13
Chloride	104(100-108)	103(99-108)	0.17	103(99-107)	103(100-108)	0.52
**Clinical measures**						
Vasopressin (n (%))	307(22.7)	29(20.0)	0.23	24(17.4)	29(21.0)	0.27
Antibiotic	1324(98.0)	140(96.6)	0.19	134(97.1)	134(97.1)	0.64
MV	1105(81.8)	119(82.1)	0.52	122(88.4)	113(81.9)	0.09

PSM, propensity score matching; RR, respiratory rate; SBP, systolic blood pressure; DBP, diastolic blood pressure; MI, myocardial infarction; AF, atrial fibrillation; CHF, chronic heart failure; CKD, chronic kidney disease; AKI, acute kidney injury; RBC, red blood cell; WBC, white blood cell; MV, mechanical ventilation.

Table S5 Clinical outcomes of young patients with sepsis (age < 60 years) after PSM

**Clinical outcomes**	All septic patients (n=140)	Non-VD Supplementation group (n=70)	VD Supplementation group (n=70)	P-value
**Primary outcomes**				
In-hospital mortality (n (%))	33(23.6)	16(22.9)	17(24.3)	0.50
28-mortality (n (%))	24(17.1)	11(15.7)	13(18.6)	0.41
90-mortality (n (%))	32(22.9)	15(21.4)	17(24.3)	0.42
**Secondary outcomes**				
Mean SAPS II	34(27-45)	34(26-44)	33(27-46)	0.17
Mean APS III	58(45-75)	58(46-82)	58.3(44-73)	0.19
Mean SOFA	7(4-9)	7(4-9)	6(4-8)	0.07
ICU LOS (days)	3.6(1.8-8.3)	3.5(1.4-9.3)	3.6(1.8-7.6)	0.77
Hospital LOS (days)	10.8(5.5-24.2)	10.8(5.0-24.6)	10.4(5.6-22.2)	0.76

PSM, propensity score matching; SAPS II, Simplified Acute Physiology Score-II; APS III, Acute Physiology Score III; SOFA, Sequential Organ Failure Assessment; LOS, lengths of stay.

Table S6 Clinical outcomes of septic patients without septic shock after PSM

**Clinical outcomes**	All septic patients (n=276)	Non-VD Supplementation group (n=138)	VD Supplementation group (n=138)	P-value
**Primary outcomes**				
In-hospital mortality (n (%))	59(21.4)	33(23.9)	26(18.8)	0.19
28-mortality (n (%))	55(19.9)	32(23.2)	23(16.7)	0.11
90-mortality (n (%))	65(23.6)	34(24.6)	31(22.5)	0.39
**Secondary outcomes**				
Mean SAPS II	41(33-49)	41(33-51)	41(34-47)	0.85
Mean APS III	52(42-73)	53(43-74)	51(41-71)	0.56
Mean SOFA	5(3-8)	6(3-8)	5(3-8)	**0.048**
ICU LOS (days)	2.3(1.4-4.9)	2.7(1.6-6.1)	2.0(1.3-3.9)	0.91
Hospital LOS (days)	9.0(5.4-16.1)	9.0(5.6-16.8)	9.1(5.3-15.3)	0.44

SAPS II, Simplified Acute Physiology Score-II; APS III, Acute Physiology Score III; SOFA, Sequential Organ Failure Assessment; LOS, lengths of stay.

Table S7 Clinical outcomes in patients with serum 25(OH)D values before and after PSM

	Before PSM			*After PSM*		
	Non-VD supplementation group (n= 21)	*VD-supplementation group (n= 12)*	*p-value*	*Non-VD supplementation group (n= 7)*	*VD-supplementation group (n= 7)*	*p-value*
serum 25(OH)D(ng/ml)	*22.50(13.00-34.50)*	*14.00(11.00-24.00)*	*< 0.001*	*16(12-22)*	*15(11-24)*	*0.64*
Clinical outcomes						
In-hospital mortality	*6(28.6)*	*3(25.0)*	*0.82*	*3(42.9)*	*1(14.3)*	*0.56*
28-day mortality	*4(19.0)*	*2(16.7)*	*0.86*	*2(28.6)*	*0(0)*	*0.46*
90-day mortality	*7(33.3)*	*4(33.3)*	*>0.90*	*3(42.9)*	*2(28.6)*	*0.59*

PSM, propensity score matching.

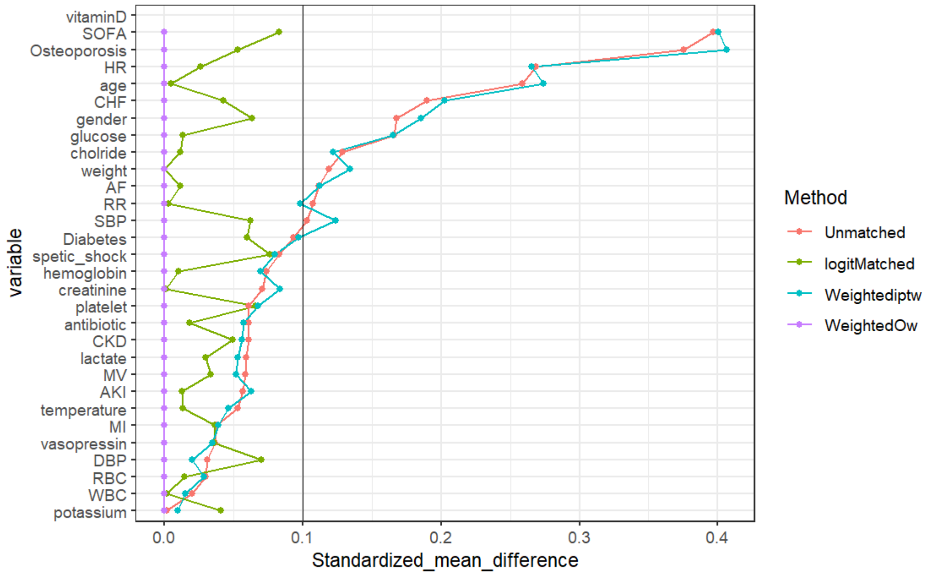

Figure S1 Standardized mean differences of baseline characteristics in unmatched, 1:1 PSM, IPTW and OW-adjusted cohort. PSM, propensity score matching; IPTW, inverse probability of treatment weighting; OW, overlap weighting.

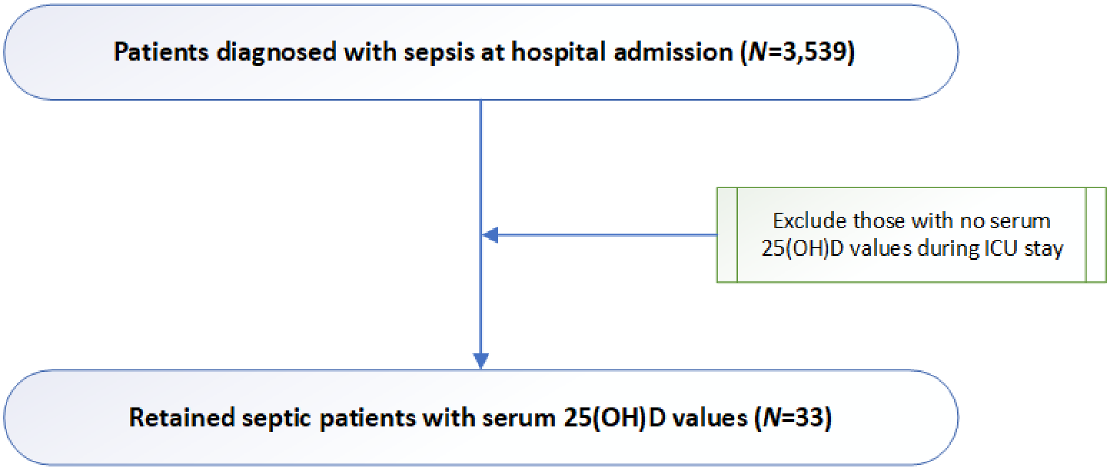

Figure S2 Flow chart of identifying patients with serum 25(OH)D values.

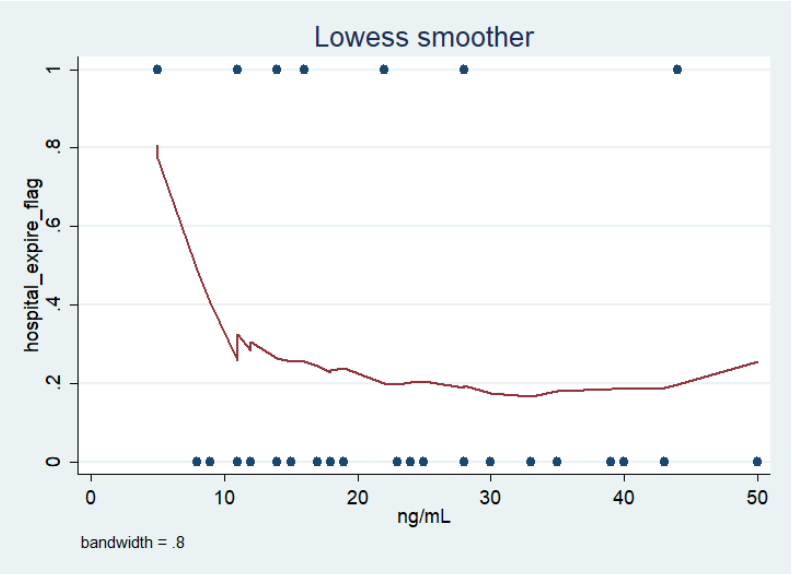

Figure S3 Association between serum 25(OH)D and in-hospital mortality in patients with serum 25(OH)D values. The y axis represents the survival probabilities of patients.
